# Drift Versus Entropic Forces in Overdamped Diffusion Through a Widening Channel

**DOI:** 10.3390/molecules30112316

**Published:** 2025-05-25

**Authors:** Michał Cieśla, Bartłomiej Dybiec, Monika Krasowska, Anna Strzelewicz

**Affiliations:** 1 Institute of Theoretical Physics and Mark Kac Center for Complex Systems Research, Jagiellonian University, St. Łojasiewicza 11, 30-348 Kraków, Poland; bartlomiej.dybiec@uj.edu.pl; 2 Faculty of Chemistry, Silesian University of Technology, Strzody 9, 44-100 Gliwice, Poland; monika.krasowska@polsl.pl (M.K.); anna.strzelewicz@polsl.pl (A.S.)

**Keywords:** overdamped diffusion, drift, ion channel, mean first passage time, numerical modeling, stochastic dynamics

## Abstract

This study examines the diffusion of spherical particles in a conical widening channel, with a focus on the effects of deterministic drift and entropic forces. Through numerical simulations, we analyze the motion of particles from a reflecting boundary to an absorbing one. Properties of diffusive motion are explored by inspection of mean squared displacement and mean first passage time. The results show that the diffusion type depends on the drift strength. Without the drift, entropic forces induce effective superdiffusion; however, the increasing drift strength can counterbalance entropic forces and shift the system to standard diffusion and then effective subdiffusion. The mean squared displacement exhibits bending points for high drift values, as predicted by one-dimensional theoretical considerations. The study underscores the importance of considering deterministic and entropic forces in confined geometries.

## 1. Introduction

The transport of single molecules, viruses, and particles passing through natural and synthetic pores exhibits peculiar features that are the subject of intense theoretical and experimental research [1,2,3,4,5,6,7,8,9,10]. Ion channels facilitate the selective passage of ions across cell membranes and play a crucial role in various physiological processes, including nerve impulse transmission, muscle contraction, and cellular signaling [11,12,13]. Ion flow across the membrane is a response to chemical or mechanical stimuli. Synthetic nanopores [14] play an important role in biotechnology [15,16] as sensors for viruses, DNA, proteins, and other molecules [17,18,19,20]. They allow for the design of sensors, the study of molecular behavior in sub-femtolitre volumes, and insight into the interactions of ions and charged molecules at the nanoscale [21,22]. The analysis of these processes attracts the interest of engineers, biologists, and physicists. Spatial confinement [23] due to the pore’s boundary and additional forces can influence diffusing particles. For instance, the dynamical properties of a system can be significantly altered by the variable cross-section of a long conical pore [24,25,26], which limits the accessible space for diffusing components and increases the hydrodynamic drag on them [27].

In recent years, numerous studies have examined the motion of particles in confined setups, revealing the intricate behaviors that arise from geometric constraints [28,29]. For example, light scattering studies have been crucial to understanding Brownian motion in restricted geometries, showing how confinement affects particle diffusion coefficients [30]. Similarly, research on the transport of nanoparticles in nanochannels has demonstrated the significant impact of confinement on axial and rotational diffusion. These studies highlight the significance of incorporating both deterministic and entropic forces in modeling particle behavior in widening channels.

The concept of entropic forces plays a crucial role in studies of diffusion in confined geometries [31,32,33,34,35]. Entropic forces arise from the constraints imposed by a channel’s geometry, affecting the available phase space for particles. In a widening channel, these forces push the particles toward the wider end, facilitating their escape. Numerical experiments show that the observed Brownian motion through the narrowing conical channel from its wider to its narrower end is effectively subdiffusive, whereas from narrower to wider, a superdiffusive character of the motion is recorded [36]. The observed subdiffusive scaling of the mean squared displacement is due to the combined action of the reflecting boundary at the beginning and the absorbing boundary at the end of the channel, as well as entropic forces, which hinder the motion towards the narrow end of the channel. Analogously, for the motion in the widening channel, effective superdiffusion is observed as entropic forces facilitate motion towards the wider end of the channel.

The study of particle motion in confined geometries has attracted significant interest due to its relevance in various scientific and engineering applications. Jacobs [37] developed a one-dimensional theoretical framework for describing diffusion in confined setups. The Fick–Jacobs approximation [38] provides a simplified description of two- or three-dimensional diffusion by reducing the problem to one dimension while accounting for the varying cross-sectional area (diameter) of the channel [39,40,41,42]. This approximation [24] has been successfully applied to various systems [34,43], including biological channels and synthetic nanopores [44]. By extending these theoretical models to include the effect of constant drift, we aim to provide a more comprehensive understanding of particle diffusion in conical channels. The diffusion in a channel involves the random motion of ions due to thermal fluctuations, which is influenced by the entropic forces arising from the confinement of the particles within the channel. In channels with non-constant diameters, the entropic repulsion due to channel walls becomes highly relevant. In addition to entropic forces, drift forces, which result from external fields or gradients, further complicate the transport process. The combination of diffusion, entropic forces, and drift leads to interesting and nontrivial behavior, which is the subject of the current research. These complex interactions can be described by methods of stochastic dynamics [45]. Numerical simulations, particularly those based on Brownian dynamics, offer a detailed and efficient way to model the transport of particles through complex geometries.

**Figure 1:**
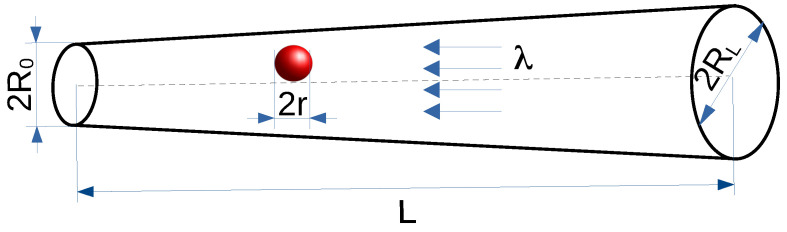
Simulation setup: The red particle of radius *r* travels through the widening channel from the reflecting boundary on the left to the absorbing one on the right. A constant drift towards the narrow end of the channel slows down the diffusive motion.

In this study, we employ numerical simulations to analyze the three-dimensional overdamped diffusion of spherical particles of radius *r* through a linearly widening conical channel under the action of thermal fluctuations, modeled by the Gaussian white noise of zero mean and $\sigma^{2}$ variance, and the deterministic (constant) drift λ. The conical channel is restricted by the reflecting boundary at its narrower end where the particle starts its motion and by the absorbing boundary at its wider, right end ($R_{0}$ is the channel radius at the narrow end, and $R_{L}$ is the channel radius at the wider end, while *L* is the channel length), see Figure 1. We focus on the exploration of how the combined action of the deterministic force and entropic forces (arising from the confinement of the particles within the channel) affects the properties of diffusion.

In our model, the drift is directed toward the reflecting boundary at the narrower, left end of the channel, while the entropic forces—arising from the channel’s widening geometry—facilitate motion toward the wider, right end. As a result, the deterministic drift can counteract the entropic forces, effectively slowing down the overall transport.

We compare the results of three-dimensional Monte Carlo simulations with the analytical one-dimensional approximation provided by the Fick–Jacobs equation. The details of numerical simulations are provided in Section 3 and Appendix A.

A key metric for analyzing diffusion occurring within the system is the particles’ mean squared displacement (MSD) [46], given by the following formula:

(1) $$\langle {\left[\vec{x}\left(t\right)-\vec{x}\left(0\right)\right]}^{2}\rangle =Dt^{\alpha },$$

where $\vec{x}\left(t\right)$ is the tracer position at time *t*, *D* is the diffusion constant, and α determines diffusion type. Averaging, in Equation (1), is performed over different independently generated trajectories, see Section 3. For normal diffusion α=1, while for subdiffusive motion, typically caused by trapping events α<1, and for superdiffusion, when, e.g., very long jumps are common α>1. Subsequently, we explore the mean first passage time (MFPT), which measures the efficiency of noise-induced escape from the channel as it is the average time needed to leave the channel for the first time.

## 2. Results and Discussion

**Figure 2:**
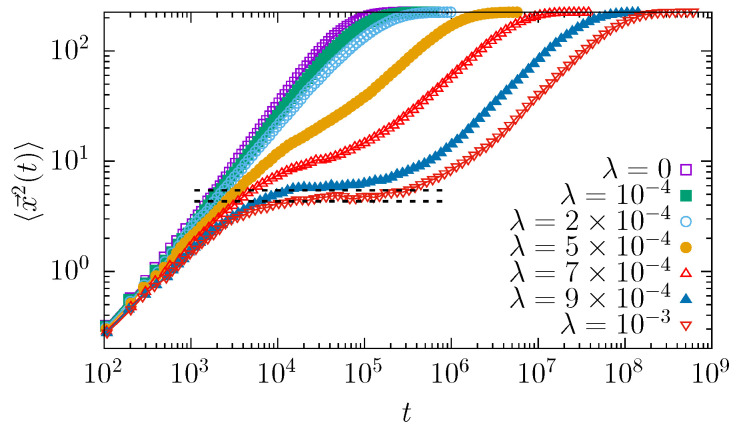
Dependence of the mean squared displacement on time for different drift strength λ. Here, r=0.0, $R_{0}=0.5$, $R_{L}=1$, L=15, and σ=0.05, see Figure 1. Dashed lines present bending points for λ=0.0009 and λ=0.001, see Table 1.

Figure 2 compares the dependence of the mean squared displacement on time for a point (r=0) particle diffusing in a three-dimensional, conical, widening channel under action of the thermal (Gaussian white) noise.

**Figure 3:**
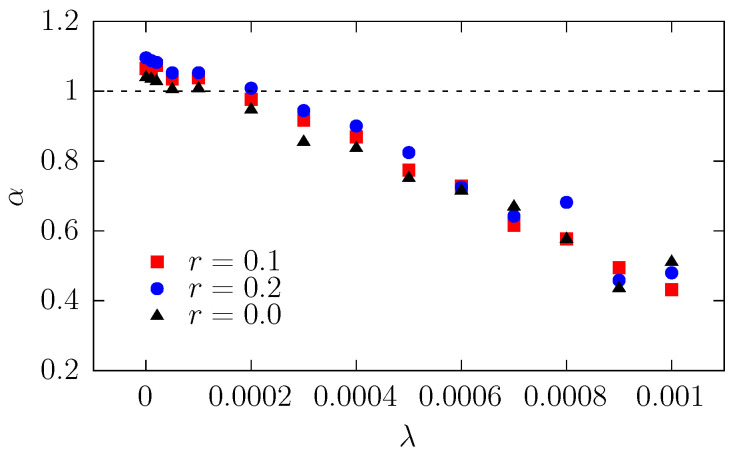
Dependence of effective diffusion exponent α on the drift strength λ for particles of different radius *r*. Dashed line corresponds to α=1, denoting a normal diffusion. Here, $R_{0}=0.5$, $R_{L}=1$, L=15, and σ=0.05, see Figure 1.

In the system studied, the type of diffusion depends on the interplay between drift and entropic forces. Figure 2 shows two interesting features that require further attention and discussion. The first is the initial change in the MSD slope for different drift strengths, while the second is the bending of the MSD curves, especially visible for higher values of λ. The first aspect is studied in terms of the exponent α—see Equation (1), which determines the type of effective diffusion observed within the channel. Its dependence on the drift strengths is shown in Figure 3.

**Figure 4:**
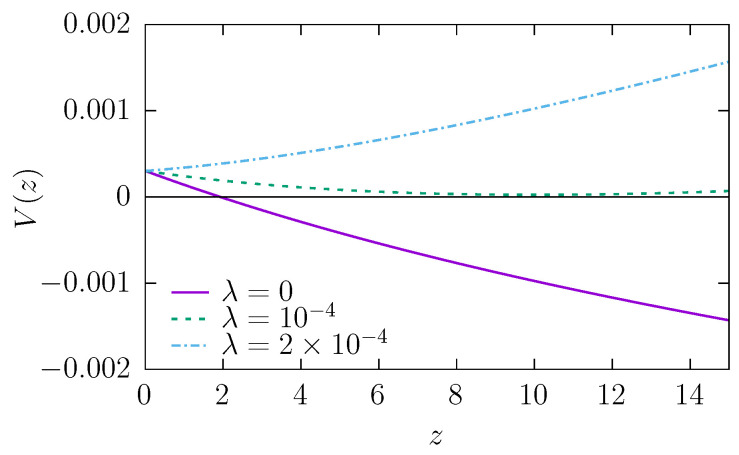
The total potential V(z), see Equation (A9), for three different values of drift strength λ. The lines were calculated assuming r=0, $R_{0}=0.5$, $R_{L}=1$, L=15, and σ=0.05, see Figure 1.

Without the deterministic drift, the parameter α is slightly larger than 1, which is caused by entropic forces due to the widening shape of the channel [36]. These forces induce a drift towards the wider end of the channel, which facilitates escape kinetics, producing effective superdiffusion. With increasing λ, as expected, the parameter α decreases because the deterministic drift pushes the particle towards the narrower part of the channel, i.e., the reflecting boundary. The α≈1 (corresponding to the normal diffusion) is observed for λ≈ 0.00006–0.0002, depending on the radius of the particles *r*—larger values are observed for larger *r* because larger objects hit the channel walls more frequently; thus, the importance of the entropic force is bigger. The influence of the entropic force and the drift on diffusion can be explored analytically using the Fick–Jacobs approximation—see Appendix A. The total potential (A9), in which a spherical particle moves, is depicted in Figure 4. The shape of the potential explains why normal diffusion is observed for λ≈0.0001. For λ≈0.0001, within the channel, the potential is almost flat (constant) and the force induced by the potential does not significantly affect the diffusive motion.

The second interesting feature of the MSD dependence on time, see Figure 2, which is especially well visible for large λ, is bending of the MSD curves. The bending point can be calculated under the assumption of quasi-stationarity [47]. For a system studied, if one replaces the absorbing boundary with the reflecting one, the system approaches a stationary state where the probability of finding a particle at a given position follows the Boltzmann–Gibbs distribution. Namely, for the three-dimensional system, it is given by

(2) $$p\left(\vec{x}\right)\propto \exp\left[-\frac{2\lambda z}{\sigma^{2}}\right],$$

and it is defined within the truncated cone

(3) $$\Omega =\{\vec{x}=\left(x,y,z\right):0⩽z⩽L\wedge x^{2}+y^{2}⩽R\left(z\right)\},$$

where R(z) is the cone radius at the distance *z*, see Equation (A7). Thus, the theoretical displacement relative to $\vec{x}\left(0\right)=\left[0,0,0\right]$ is

(4) $$MSD={\;\int \int \int \;}_{\Omega }p\left(x,y,z\right)\left(z^{2}+x^{2}+y^{2}\right)dxdydz,$$

where integration is performed over the truncated cone, see Figure 1. For σ=0.05, one gets the values included in Table 1.

In Figure 2, the dashed lines correspond to the above-calculated values, see Table 1. For λ=0.0009 and λ=0.001, they fit very well the flat regions in the MSD dependence on time. It suggests that for strong enough drift (λ), the system has enough time to equilibrate before the escape events are recorded. Consequently, it is well approximated by the stationary density given by Equation (2) and the MSD bends at values predicted by Equation (4). Alternatively, it is possible to calculate the MSD from the one-dimensional model, see Equation (A9). However, in such a case, all integrals are one-dimensional and the potential includes the entropic part, see Equation (A9). Such calculations give similar results to the one included in Table 1.

**Figure 5:**
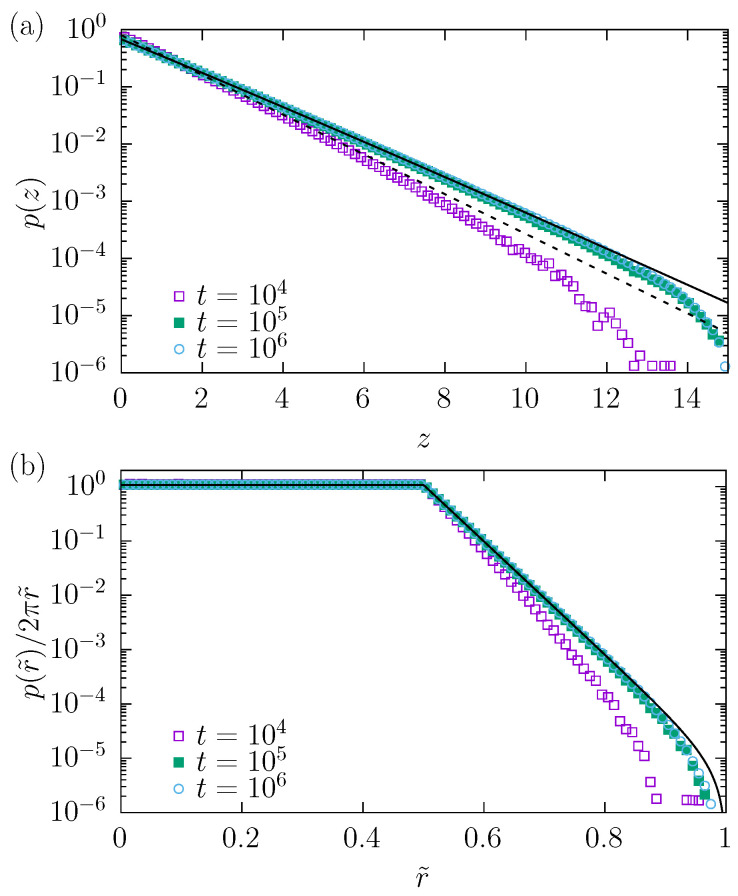
Marginal p(z) and $p\left(\tilde{r}\right)=p\left(\sqrt{x^{2}+y^{2}}\right)$ densities at various time instants $t\in \{10^{4},10^{5},10^{6}\}$ for $\lambda =10^{-3}$ with σ=0.05. (**a**) Points depict the dependence of p(z) estimated from simulations. The solid line shows the theoretical marginal density p(z), while the dashed line shows the slope predicted by the Boltzmann–Gibbs distribution, see Equation (2). (**b**) Re-normalized marginal density $p\left(\tilde{r}\right)/2\pi \tilde{r}$. The solid line shows the re-normalized theoretical marginal density $p(\tilde{r})$, while points represent results of computer simulations.

The problem of equilibration is studied in more detail in Figure 5. It shows the numerically estimated (three-dimensional diffusion) marginal densities of p(z) (Figure 5a) at various time moments along with the theoretical formula (solid line) and the slope given by Equation (2) (dashed line). Similarly, in Figure 5b, we show the numerically estimated marginal densities of $p(\tilde{r})$, where $\tilde{r}=\sqrt{x^{2}+y^{2}}$, along with the theoretical shape (solid line). These theoretical marginal densities $p(\tilde{r})$ and p(z) have been calculated by integrating Equation (2) over *z*, or *x* and *y*, respectively. Comparison of theoretical and numerically estimated marginal densities indicates that the density $p(\vec{x})$ can be very well approximated by Equation (2). This indicates that for strong enough drift, the escape process is slow enough to ensure the system’s equilibration. For clarity, in Figure 5b, marginal densities $p(\tilde{r})$ are renormalized by the $2\pi \tilde{r}$ factor to show that the points are uniformly distributed over the circle $0<\tilde{r}<R_{0}=1/2$, while for $R_{0}<\tilde{r}⩽R_{L}$, the density decays to zero. Finally, in the limit of λ→∞, all particles are uniformly placed on the entry of the channel (circle of radius $R_{0}$), and the theoretical value of MSD tends to 1/8.

### Mean First Passage Time

The mean first passage time is the average time that a particle needs to leave the channel for the first. On the one hand, it can be estimated from direct simulations of three-dimensional diffusion. On the other hand, it can be calculated from analytical formulas.

For a one-dimensional case and a particle starting near a reflective boundary at z=0 and moving in the potential V(z) toward the absorbing boundary at z=b, the mean first passage time reads ([48], Equation (5.5.23))

(5) $$T=\frac{2}{\sigma^{2}}\int_{0}^{b}dz^{\prime }\int_{a}^{z^{\prime }}\exp\left[\frac{2V\left(z^{\prime }\right)-2V\left(z^{\prime \prime }\right)}{\sigma^{2}}\right]dz^{\prime \prime }.$$

**Figure 6:**
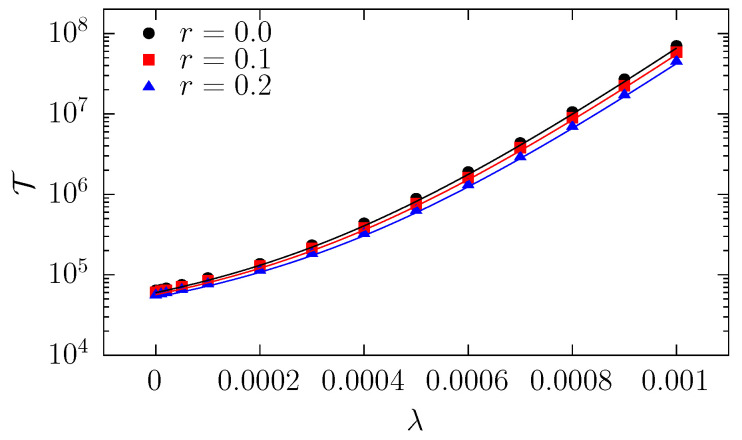
The dependence of the mean first passage time on drift strength λ for particles characterized by various radii *r*. The plot compares numerical results (points) with analytical ones (solid lines) based on Equation (5) with V(z) given by Equation (A9). Simulations were performed for $R_{0}=0.5$, $R_{L}=1$, L=15, and σ=0.05, see Figure 1.

The numerically estimated values of the mean passage time are collected in Figure 6. As expected, the mean passage time increases with increasing drift strength and is slightly lower for larger particles due to a higher entropic force that speeds up the escape process. It is notable that the measured values, in general, agree with the theoretical predictions, see Equations (5) and (A9); however, the former are slightly larger. This is because the one-dimensional theoretical analysis does not fully account for collisions with channel boundaries. To confirm this, we checked the passage times for a narrower channel, and there, the MFPTs were higher.

**Figure 7:**
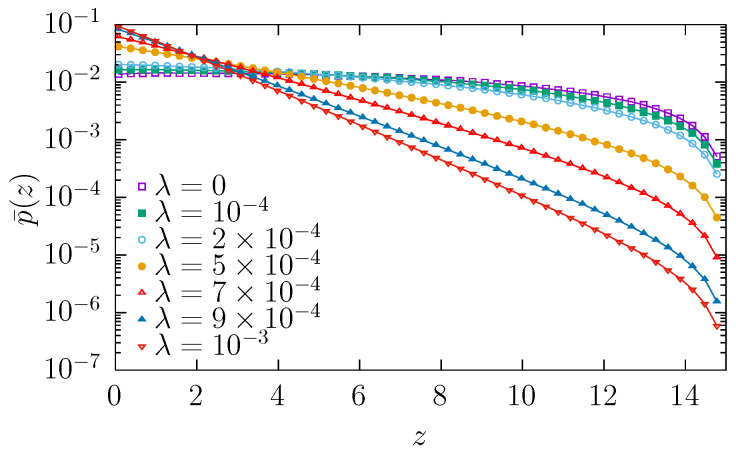
Fraction of time $\bar{p}\left(z\right)$ spent at given position *z*. Results were collected for r=0, $R_{0}=0.5$, $R_{L}=1$, L=15, and σ=0.05, see Figure 1.

Finally, in Figure 7, the fraction of time $\bar{p}\left(z\right)$ that the particle spends at a given point *z* is depicted. This figure, in accordance with Figure 5, confirms that escaping particles spend the majority of time in the vicinity of the reflecting boundary. Moreover, with the increasing drift strength, the fraction of time spent in the neighborhood of the reflecting boundary increases.

## 3. Materials and Methods

A spherical particle of radius *r* diffuses in the three-dimensional, conical widening channel. A particle starts its motion from the center of the left, narrower end of the channel, $\vec{x}\left(0\right)=\left[0,0,0\right]$, see Figure 1. The *z*-axis coincides with the channel axis, while the x,y axes are perpendicular to it. The origin is placed in the middle of the channel entry. The particles’ movements are also subjected to a constant drift that pushes them to the left and hinders their motion to the right. The motion of the tracer is described by the overdamped Langevin equation

(6) $$\frac{d\vec{x}}{dt}=\sigma \vec{\xi }\left(t\right)-\vec{\lambda },$$

where $\vec{x}$ is the particle position, $\vec{\xi }\left(t\right)$ is a three-dimensional Gaussian white noise, and $\vec{\lambda }$ is the constant drift. Equation (6) can be approximated by [49,50]

(7) $$\vec{x}\left(t+1\right)=\vec{x}\left(t\right)+\sigma \vec{\xi }\left(t\right)-\vec{\lambda },$$

where *t* is time, $\vec{\lambda }=\left[0,0,\lambda \right]$ is the drift, and components of the three-dimensional Gaussian white noise $\vec{\xi }\left(t\right)$ follow $\langle \xi_{i}\left(t\right)\rangle =0$ and $\langle \xi_{i}\left(t\right)\xi_{j}\left(s\right)\rangle =\delta_{ij}\delta \left(t-s\right)$, where $\delta_{ij}$ is a Kronecker’s delta, while δ(t−s) is the Dirac’s delta and σ scales the noise strength. Thus, components of $\vec{\xi }$ were drawn from the normal distribution of zero mean and unit variance N(0,1) using the Box–Muller algorithm. The displacement is accepted only if $\vec{x}\left(t+1\right)$ is inside the channel. Otherwise, $\vec{x}\left(t+1\right)=\vec{x}\left(t\right)$. Equation (7) represents the Euler–Maruyama approximation [49,50] adopted to the situation when time is measured in the number of jumps. In this manner, assuming that $\vec{x}\left(0\right)=\left[0,0,0\right]$, we generate a single trajectory $\left\{\vec{x}\left(0\right),\vec{x}\left(1\right),\vec{x}\left(2\right),\ldots \right\}$. The trajectory generation is stopped when the center of a tracer reaches the wider right end of the channel; that is, the *z* component of the tracer position $\vec{x}$ exceeds the channel length *L*, i.e., z(t)⩾L. The parameter σ was set to 0.05, which gives a reasonable compromise between trajectory length and duration of simulation (escape time). Furthermore, this particular value was used in our previous studies on diffusion transport within a channel [36,51,52]. The parameter σ is directly related to the variance of random kicks acting on a particle. Thus, at the microscopic level, it is related to the system temperature and the diffusion constant. The time at which, for the first time, z(t)⩾L is the first passage time.

The channel shape, see Figure 1, is determined by three parameters $R_{0},R_{L}$, and *L*, which have been adjusted to $R_{0}=0.5$, $R_{L}=1$, and L=15. These dimensions, together with the radius of the tracer *r* (0⩽r⩽0.2), were chosen to resemble the relative dimensions of the real and artificial channels used in the context of biophysical studies [14,53].

For a particle traveling within the channel, there are two natural time scales. One corresponds to the noise-induced escape from a tube of length *L* and is equal to $L^{2}/\sigma^{2}=9000$. The second one is associated with the time of the deterministic travel along the whole channel, which is determined by the drift λ. It reads L/λ, and for $\lambda \in (0,10^{-3}]$, it is larger than 15,000. Consequently, both timescales are a few orders of magnitude higher than the integration time step, see Equation (7). The particle trajectory $\vec{x}$ is a random process. Consequently, exploration of diffusion in the conical channel requires ensemble averaging over realizations. The presented results, see Section 2, are typically based on the simulation of $10^{4}$ independent trajectories. In addition to trajectories, we recorded the number of time steps needed to exit the channel. From the ensemble of trajectories, we have estimated various characteristics of the diffusion, including the mean squared displacement, see Equation (1), and the mean first passage time. To calculate the MSD at a given time *t*, we perform ensemble averaging over the positions of particles recorded at that time across multiple independent trajectories. To estimate the MFPT, we compute the average of the first passage times obtained from each trajectory. The first passage time τ is defined as the time at which a trajectory exits the channel for the first time, i.e., z(τ)⩾L.

## 4. Conclusions

The main conclusion of this study is that the one-dimensional Fick–Jacobs approximation accurately predicts the mean first passage times of particles diffusing through a three-dimensional, widening channel under the influence of an external drift. Slight discrepancies arise due to the finite size of the channel and the presence of boundaries, which restrict certain random movements in our model. As a result, the mean first passage times are slightly larger than those predicted by the one-dimensional theoretical approximation. The second key observation is that the mean squared displacement reveals that the effective diffusion can exhibit superdiffusive, normal, or subdiffusive signature, depending on the relative strength of the external drift and the entropic forces induced by the channel’s geometry. This was partially expected, because in the absence of drift, entropic forces induce effective superdiffusion by facilitating particle escape toward the wider end of the channel. On the other hand, theoretical predictions suggest only a slight modification of the diffusion coefficient [31,38,40], rather than a change in the diffusion type. However, recent studies have shown that effective diffusion in such systems can indeed change due to the influence of entropic forces and reflecting boundaries [36,52]. As the drift strength increases, the diffusion transitions from superdiffusive to normal and eventually to subdiffusive behavior as the drift pushes particles toward the narrower, reflecting boundary. For sufficiently strong drift, the system approaches a quasi-stationary state that follows the standard Boltzmann–Gibbs distribution. This situation is analogous to a constant gravitational field, which creates a gradient in air density—particles tend to accumulate near the reflecting boundary in a manner similar to how air molecules concentrate near the Earth’s surface. Numerical simulations confirm that escaping particles spend most of their time near the reflecting boundary, and this tendency becomes more pronounced as the drift strength increases.

This research deepens our understanding of diffusion processes in confined geometries by examining the relationship between full three-dimensional descriptions and reduced, approximate models. Our findings highlight the critical role of both deterministic and entropic forces in accurately modeling and predicting particle behavior in complex, constrained environments, particularly biological channels and synthetic nanopores.

## Figures and Tables

**Table 1:** Theoretical values of MSD, see Equation (4), for stationary probability distribution (2) calculated for σ=0.05, $R_{0}=0.1$, $R_{L}=1$, and L=15, see Figure 1.

***λ***	0.0001	0.0002	0.005	0.0007	0.0009	0.001
**MSD**	77.71	56.79	18.56	9.42	5.45	4.33

## Data Availability

Data from numerical simulation are available through the link https://doi.org/10.57903/UJ/BJYPLZ.

## Abbreviations

The following abbreviations are used in this manuscript:

MSD	mean squared displacement
MFPT	mean first passage time

## Appendix A. Fick–Jacobs Description of Entropic Forces

The three-dimensional diffusive motion in a conical channel can be effectively approximated by a one-dimensional description [37]. The Fick–Jacobs equation [38]

(A1) $$\frac{\partial }{\partial t}P\left(z,t\right)=D\frac{\partial }{\partial z}A\left(z\right)\frac{\partial }{\partial z}\frac{P(z,t)}{A(z)}$$

describes the diffusion in a rotationally symmetric channel of area A(z) (diameter in two dimensions), where *z*-axis is the channel axis, see Figure 1. Using the association [38]

(A2) $$A\left(z\right)⟷\exp\left[-\frac{V_{S}\left(z\right)}{D}\right]$$

Equation (A1) can be transformed into the one-dimensional Smoluchowski equation [48]. After inserting (A2) into Equation (A1), one obtains

(A3) $$\frac{\partial }{\partial t}P\left(z,t\right)=\frac{\partial }{\partial z}\left[V_{S}^{\prime }\left(z\right)P\left(z,t\right)\right]+D\frac{\partial^{2}}{\partial z^{2}}P\left(z,t\right),$$

which is associated with the following Langevin equation

(A4) $$\frac{dz}{dt}=-V_{S}^{\prime }\left(z\right)+\sqrt{2D}\xi \left(t\right),$$

where ξ(t) is the standard Gaussian white noise (〈ξ(t)〉=0 and (〈ξ(t)ξ(s)〉=δ(t−s)). For the setup described by Equation (A3), formulas for the mean first passage time [48] can be used, while from Equation (A2), the entropic potential can be obtained

(A5) $$V_{S}\left(z\right)=-D\ln A\left(z\right),$$

where $D=\sigma^{2}/2$, see Equation (7). Equation (A4) can be studied trajectory-wise [49,50], while Equation (A3) can be treated by general methods [48,54].

In the case studied here, on the one hand, the entropic potential reads

(A6) $$V_{S}\left(z\right)=-\frac{\sigma^{2}}{2}\ln\left[\pi {\left(R(z)-r\right)}^{2}\right],$$

where R(z) is the channel radius at the distance *z* (measured from the left)

(A7) $$R\left(z\right)=R_{0}+\frac{R_{L}-R_{0}}{L}z,$$

*z*-axis coincides with the axis of symmetry of the channel, while *r* is a particle radius. On the other hand, the potential $V_{\lambda }$ corresponding to the external drift is

(A8) $$V_{\lambda }\left(z\right)=\lambda z.$$

Thus, the total potential is the sum of these two components

(A9) $$V\left(z\right)=V_{S}\left(z\right)+V_{\lambda }\left(z\right).$$

## References

[1] Kasianowicz J.J., Brandin E., Branton D., Deamer D.W. (1996). Characterization of individual polynucleotide molecules using a membrane channel. Proc. Nat. Acad. Sci. USA.

[2] Bayley H., Martin C.R. (2000). Resistive-Pulse Sensing: From Microbes to Molecules. Chem. Rev..

[3] Uram J.D., Ke K., Hunt A.J., Mayer M. (2006). Submicrometer Pore-Based Characterization and Quantification of Antibody–Virus Interactions. Small.

[4] Dekker C. (2007). Solid-state nanopores. Nat. Nanotechnol..

[5] Iqbal S.M., Akin D., Bashir R. (2007). Solid-state nanopore channels with DNA selectivity. Nat. Nanotechnol..

[6] Makhnovskii Y., Berezhkovskii A., Zitserman V. (2010). Diffusion in a tube of alternating diameter. Chem. Phys..

[7] Bezrukov S.M., Schimansky-Geier L., Schmid G. (2014). Brownian motion in confined geometries. Eur. Phys. J. Spec. Top..

[8] Liu L., Cherstvy A.G., Metzler R. (2017). Facilitated Diffusion of Transcription Factor Proteins with Anomalous Bulk Diffusion. J. Phys. Chem. B..

[9] Horne R.I., Sandler S.E., Vendruscolo M., Keyser U.F. (2025). Detection of protein oligomers with nanopores. Nat. Rev. Chem..

[10] Kalinay P. (2025). Effective dynamics of a particle diffusing in time-dependent confinement. Phys. Rev. E.

[11] Hille B. (1978). Ionic channels in excitable membranes. Current problems and biophysical approaches. Biophys. J..

[12] Hille B. (2001). Ion Channels of Excitable Membranes.

[13] Thompson M.J., Baenziger J.E. (2020). Ion channels as lipid sensors: From structures to mechanisms. Nat. Chem. Biol..

[14] Diederichs T., Ahmad K., Burns J.R., Nguyen Q.H., Siwy Z.S., Tornow M., Coveney P.V., Tampé R., Howorka S. (2021). Principles of small-molecule transport through synthetic nanopores. ACS Nano.

[15] Cherf G.M., Lieberman K.R., Rashid H., Lam C.E., Karplus K., Akeson M. (2012). Automated forward and reverse ratcheting of DNA in a nanopore at 5-Å precision. Nat. Biotechnol..

[16] Chien C.C., Shekar S., Niedzwiecki D.J., Shepard K.L., Drndić M. (2019). Single-Stranded DNA Translocation Recordings through Solid-State Nanopores on Glass Chips at 10 MHz Measurement Bandwidth. ACS Nano.

[17] Venkatesan B.M., Bashir R. (2011). Nanopore sensors for nucleic acid analysis. Nat. Nanotechnol..

[18] Wanunu M. (2012). Nanopores: A journey towards DNA sequencing. Phys. Life Rev..

[19] Xue L., Yamazaki H., Ren R., Wanunu M., Ivanov A.P., Edel J.B. (2020). Solid-state nanopore sensors. Nat. Rev. Mater..

[20] Akhtarian S., Miri S., Doostmohammadi A., Brar S.K., Rezai P. (2021). Nanopore sensors for viral particle quantification: Current progress and future prospects. Bioengineered.

[21] Tagliazucchi M., Szleifer I. (2015). Transport mechanisms in nanopores and nanochannels: Can we mimic nature?. Mater. Today.

[22] Sirkin Y.P., Tagliazucchi M., Szleifer I. (2020). Transport in nanopores and nanochannels: Some fundamental challenges and nature-inspired solutions. Mater. Today Adv..

[23] Gubbiotti A., Chinappi M., Casciola C.M. (2019). Confinement effects on the dynamics of a rigid particle in a nanochannel. Phys. Rev. E.

[24] Berezhkovskii A.M., Pustovoit M.A., Bezrukov S.M. (2007). Diffusion in a tube of varying cross section: Numerical study of reduction to effective one-dimensional description. J. Chem. Phys..

[25] Rubi J.M. (2019). Entropic diffusion in confined soft-matter and biological systems. Europhys. Lett..

[26] Dagdug L., Berezhkovskii A.M., Bezrukov S.M. (2023). Diffusion Resistance of Segmented Channels. J. Phys. Chem. B.

[27] Yang X., Liu C., Li Y., Marchesoni F., Hänggi P., Zhang H.P. (2017). Hydrodynamic and entropic effects on colloidal diffusion in corrugated channels. Proc. Natl. Acad. Sci. USA.

[28] Li Y., Mei R., Xu Y., Kurths J., Duan J., Metzler R. (2020). Particle dynamics and transport enhancement in a confined channel with position-dependent diffusivity. New J. Phys..

[29] Kubala P., Cieśla M., Dybiec B. (2021). Diffusion in crowded environments: Trapped by the drift. Phys. Rev. E.

[30] Lançon P., Batrouni G., Lobry L., Ostrowsky N. (2002). Brownian walker in a confined geometry leading to a space-dependent diffusion coefficient. Physica A.

[31] Reguera D., Rubí J.M. (2001). Kinetic equations for diffusion in the presence of entropic barriers. Phys. Rev. E.

[32] Burada P.S., Schmid G., Talkner P., Hänggi P., Reguera D., Rubí J.M. (2008). Entropic particle transport in periodic channels. BioSystems.

[33] Riefler W., Schmid G., Burada P.S., Hänggi P. (2010). Entropic transport of finite size particles. J. Condens. Matter Phys..

[34] Reguera D., Luque A., Burada P.S., Schmid G., Rubí J.M., Hänggi P. (2012). Entropic Splitter for Particle Separation. Phys. Rev. Lett..

[35] Berezhkovskii A.M., Bezrukov S.M. (2014). On the applicability of entropy potentials in transport problems. Eur. Phys. J. Spec. Top..

[36] Cieśla M., Dybiec B., Krasowska M., Strzelewicz A. (2025). Effective anomalous diffusion in a conical channel. Chaos.

[37] Jacobs M.H. (1967). Diffusion Processes.

[38] Zwanzig R. (1992). Diffusion past an entropy barrier. J. Phys. Chem..

[39] Kalinay P., Percus J. (2005). Projection of two-dimensional diffusion in a narrow channel onto the longitudinal dimension. J. Phys. Chem..

[40] Kalinay P., Percus J. (2006). Corrections to the Fick-Jacobs equation. Phys. Rev. E.

[41] Mangeat M., Guérin T., Dean D.S. (2017). Dispersion in two dimensional channels—The Fick–Jacobs approximation revisited. J. Stat. Mech..

[42] Berezhkovskii A.M., Dagdug L., Bezrukov S.M. (2017). First passage, looping, and direct transition in expanding and narrowing tubes: Effects of the entropy potential. J. Chem. Phys..

[43] Slater G.W., Guo H.L., Nixon G.I. (1997). Bidirectional transport of polyelectrolytes using self-modulating entropic ratchets. Phys. Rev. Lett..

[44] Berezhkovskii A., Dagdug L., Bezrukov S.M. (2014). Discriminating between Anomalous Diffusion and Transient Behavior in Microheterogeneous Environments. Biophys. J..

[45] Gordon D., Chen R., Ho J., Coote M., Chung S. (2012). Rigid Body Brownian Dynamics as a Tool for Studying Ion Channel Blockers. J. Phys. Chem. B.

[46] Metzler R., Klafter J. (2000). The random walk’s guide to anomalous diffusion: A fractional dynamics approach. Phys. Rep..

[47] Šiler M., Ornigotti L., Brzobohatý O., Jákl P., Ryabov A., Holubec V., Zemánek P., Filip R. (2018). Diffusing up the Hill: Dynamics and Equipartition in Highly Unstable Systems. Phys. Rev. Lett..

[48] Gardiner C.W. (2009). Handbook of Stochastic Methods for Physics, Chemistry and Natural Sciences.

[49] Higham D.J. (2001). An algorithmic introduction to numerical simulation of stochastic differential equations. SIAM Rev..

[50] Mannella R. (2002). Integration of Stochastic Differential Equations on a Computer. Int. J. Mod. Phys. C.

[51] Cieśla M., Dybiec B., Krasowska M., Siwy Z., Strzelewicz A. (2024). Numerical Modeling of Anisotropic Particle Diffusion through a Cylindrical Channel. Molecules.

[52] Strzelewicz A., Cieśla M., Dybiec B., Krasowska M. (2025). Modeling Diffusion of Elongated Particles Through a Narrowing Channel. Entropy.

[53] Schiel M., Siwy Z.S. (2014). Diffusion and trapping of single particles in pores with combined pressure and dynamic voltage. J. Phys. Chem. C.

[54] van Kampen N. (2007). Stochastic Processes in Physics and Chemistry.

